# Simultaneous Detection of α-Fetoprotein and Carcinoembryonic Antigen Based on Si Nanowire Field-Effect Transistors

**DOI:** 10.3390/s150819225

**Published:** 2015-08-05

**Authors:** Kuiyu Zhu, Ye Zhang, Zengyao Li, Fan Zhou, Kang Feng, Huiqiang Dou, Tong Wang

**Affiliations:** 1 Nanjing Medical University, Nanjing 210029, China; E-Mails: kuiyuzhu4@163.com (K.Z.); zengyaoli44@163.com (Z.L.); fanzhou4@163.com (F.Z.); kangfeng44@163.com (K.F.); 2 Wuxi People’s Hospital, Wuxi 214023, China; E-Mails: yezhang4555@163.com (Y.Z.); douhuiqiang4@163.com (H.D.)

**Keywords:** primary hepatic carcinoma, silicon nanowire field-effect transistor, α-fetoprotein, carcinoembryonic antigen, polydimethylsiloxane microfluidic channel

## Abstract

Primary hepatic carcinoma (PHC) is one of the most common malignancies worldwide, resulting in death within six to 20 months. The survival rate can be improved by effective treatments when diagnosed at an early stage. The α-fetoprotein (AFP) and carcinoembryonic antigen (CEA) have been identified as markers that are expressed at higher levels in PHC patients. In this study, we employed silicon nanowire field-effect transistors (SiNW-FETs) with polydimethylsiloxane (PDMS) microfluidic channels to simultaneously detect AFP and CEA in desalted human serum. Dual-channel PDMS was first utilized for the selective modification of AFP and CEA antibodies on SiNWs, while single-channel PDMS offers faster and more sensitive detection of AFP and CEA in serum. During the SiNW modification process, 0.1% BSA was utilized to minimize nonspecific protein binding from serum. The linear dynamic ranges for the AFP and CEA detection were measured to be 500 fg/mL to 50 ng/mL and 50 fg/mL to 10 ng/mL, respectively. Our work demonstrates the promising potential of fabricated SiNW-FETs as a direct detection kit for multiple tumor markers in serum; therefore, it provides a chance for early stage diagnose and, hence, more effective treatments for PHC patients.

## 1. Introduction

Primary hepatic carcinoma (PHC) is currently the fifth most common malignancy worldwide, leading to death within six to 20 months [1,2]. PHC frequently arises in hepatocellular diseases, including cirrhosis caused by hepatitis B or C, alcoholic cirrhosis and autoimmune hepatitis [3,4]. Several clinical symptoms, such as weight loss, weakness and epigastric pain, have been employed to diagnose PHC along with hepatic scanning, histology and serum markers [5]. However, if patients are diagnosed at an early stage, the survival rate can be further improved by more effective treatments, such as surgical resection, tumor ablation or liver transplantation. With the development of diagnostic methods, patients are often diagnosed without clinical symptoms. For example, tumor marker analysis has become a new method in diagnosing metastases, aiding clinical decision-making [6]. α-fetoprotein (AFP) and carcinoembryonic antigen (CEA) have been frequently suggested as two valuable adjuncts in cancer diagnosis according to their high expression levels associated with cancer [7,8]. Detection methods, such as external photo-scanning after labeling specific antigens, polymerase chain reaction and immunoassay, are commonly combined with AFP and CEA to diagnose PHC [9,10,11,12]. Since the cut-off values of AFP and CEA are low, the usefulness of the detection methods above is limited to diagnosing asymptomatic cancer at an early stage; hence, more effective, timesaving methods with high sensitivity and specificity are needed.

Recently, biosensors have been proposed as promising analytical devices and employed in different fields, such as clinical diagnostics, both with or without labeling [13]. Label-free biosensors based on silicon nanowire field-effect transistors (SiNW-FETs) have been successful applied in cancer diagnosis with excellent specificity and high sensitivity [14]. SiNW-FET biosensors, consisting of source, drain and gate electrodes, detect the changes of charge density on the surface of the SiNW caused by the intrinsic charge of adsorbed biomolecules [15]. Therefore, it has already been widely used as a high performance biosensor in the fields of biological research, including detection of proteins, DNA sequences, small molecules, cancer biomarkers and viruses [16]. Moreover, polydimethylsiloxane (PDMS) has been extensively used to manufacture microfluidic channels placed on the surfaces of biosensors, allowing controllable solution flow over the nanowires [17]. The employment of PDMS also improves the reliability to simultaneously detect multiple cancer biomarkers. However, to date, few studies were performed on the measurements of AFP and CEA simultaneously using SiNW-FET for the early-stage diagnosis of PHC.

In this study, we fabricated a SiNW-FET biosensor using top-down microfabrication technology. The dual-channel PDMS setup was designed to modify SiNW by both AFP and CEA antibodies as two probes, while a single-channel PDMS setup was employed to achieve the simultaneous detection of AFP and CEA by biosensor.

## 2. Experimental Section

### 2.1. Materials

Four-inch silicon-on-insulator wafers (SOI, p-type with resistivity of 10–20 Ω∙cm; device layer: 190 nm; buried oxidation layer: 375 nm) were purchased from Shanghai Simgui Corporation (Shanghai, China). AFP, CEA and their antibodies were purchased from Fitzgerald Industries International (Acton, USA). PBS of pH 7.4 was made in our laboratory using 137 mM NaCl, 8.1 mM Na_2_HPO_4_·12H_2_O, 2.7 mM KCl and 1.5 mM KH_2_PO_4_. Bovine serum albumin (BSA) and human serum was purchased from Sigma-Aldrich. (3-aminopropyl) triethoxysilane (APTES), glutaraldehyde and tetramethylammonium hydroxide (TMAH) were purchased from Alfa-Aesar. PDMS was purchased from Dow Corning Corporation. The deionized water (DI) (R ≥ 18.2 MΩ cm) was produced by a Millipore system.

### 2.2. Fabrication of SiNW-FET Chips

**Figure 1:**
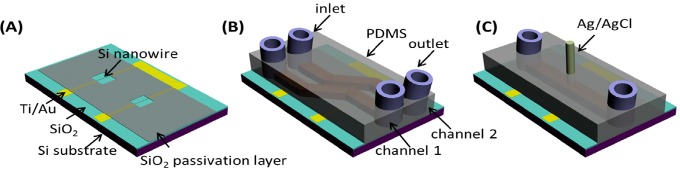
Schematic illustrations for the silicon nanowire field-effect transistor (SiNW-FET) chip (**A**); microfluidic channels during antibody modifications (dual-channel); (**B**) and biosensing measurements (single channel) (**C**).

In this work, we fabricated the SiNW-FET chips using top-down microfabrication technology, which possessed the advantages of large scale, high consistency, *etc*. Figure 1A depicts the basic structure of the SiNW-FET consisting of SiNWs, metal contacts and passivation layer. The general fabrication route for the SiNW-FETs was as follows. Firstly, a thermal oxidation process was applied to thin down the thickness of the device layer of SOI wafers to around 45 nm. Then, UV photolithography was employed to define the patterns of contact electrodes on the surface of the substrates. Twenty nanometers of Ti and 150 nm of Au were deposited on the substrates by magnetron sputtering followed by the lift-off process. The Si nanowire pattern with an initial 200 nm width was sequentially defined by electron-beam lithography (EBL) followed by deposition of 50 nm Cr as the sacrifice layer. The width of Si nanowire was then decreased to around 100 nm by TMAH wet etching. Although EBL technology can achieve a width of Si nanowire as small as 10 nm, we found in our experiments that there existed some defects, inducing charge trapping if the nanowire was directly patterned to 100 nm using EBL followed by reactive ion etching (RIE). Wet etching with TMAH may significantly eliminate this issue. After removal of Cr layers by Cr etchant, these devices were thermally annealed at 750 °C for 90 s in N_2_ atmosphere to establish ohmic contacts between electrodes and silicon. To decrease the signal interference from metal contacts during biosensing measurements in a liquid environment, a passivation layer composed of 500 nm SiO_2_ thin film was further deposited on the SOI wafer. The SiO_2_ thin film was first deposited by the low-pressure chemical vapor deposition (LPCVD) method, followed by UV-lithography and reactive ion etching (RIE) processes to open nanowire and metal contact zones, as shown in Figure 1A.

### 2.3. Fabrication of PDMS Microfluidic Channels

Microfluidic channels covering the SiNW-FETs were fabricated with PDMS. Firstly, channel molds were patterned on Si wafers by UV-lithography and inductively-coupled plasma (ICP) etching (to etch around a 500-μm depth in Si). After that, the Si molds were processed in fluoroalkyl silane to form hydrophobic surfaces, which facilitated the removal of PDMS from Si molds. The mixture of PDMS prepolymer and its curing agent (10:1) was then cast onto the Si molds and solidified at 90 °C. After peeling off from the Si molds, the PDMS microfluidic channels were ready for integration with SiNW-FETs. In this work, two kinds of PDMS microfluidic channels were designed: dual channels for SiNW surface modifications (Figure 1B) and a single channel for biosensing measurements (Figure 1C).

### 2.4. Modification of SiNW-FET with AFP and CEA Antibodies

In this work, we proposed to detect AFP and CEA on a single SiNW-FET chip simultaneously. Dual-channel PDMS was first utilized to achieve the selective functionalization of SiNWs with two different AFP and CEA antibodies. FET biosensor chips were initially immersed in 2% APTES ethanol solution, which reacted with the hydroxyl groups on the oxidation layer of SiNW surfaces for 45 min. Then, the chips were heated at 150 °C for 1 h to remove unmodified APTES molecules before immersing in 2.5% glutaraldehyde aqueous solution. Dual-channel PDMS was then integrated with the FET biosensor, so that AFP and CEA antibodies (both 10 μg/mL) in PBS buffer (pH 7.4) can be injected through individual channels and immobilized on the silicon surfaces by covalent bonding. After the modification of AFP and CEA antibodies, 0.1% BSA blocking agent was then injected to block the unspecific binding sites. After that, dual-channel PDMS was removed, and single-channel PDMS was mounted on the chip surface. All of the modified biosensor chips were kept in 4 °C before biosensing measurements.

### 2.5. Measurements of the Biosensors

Single-channel PDMS was integrated on the SiNW-FET chip and utilized to detect AFP and CEA in desalted human serum, which was injected by a syringe pump with a flow speed of 20 μL/min. The desalting treatments of the human serum followed a previously established method by Zheng *et al.* [18] with slight modifications. In detail, human serum with certain concentrations of AFP and CEA was first transferred to a microcentrifuge filter (Sartorius Stedim Biotech, 3000 MWCO). The desalting process was accomplished by centrifuging at around 5000× *g* for 90 min at room temperature. The desalted portion of serum was then diluted back to the original protein concentrations with buffer solution composed of 1 μM phosphate buffer and 2 μM KCl. When performing biosensing measurements, an Ag/AgCl reference electrode was mounted on the single-channel PDMS as the liquid gate. All of the measurements proceeded at room temperature, and a Keithley 4200 parameter analyzer was employed to analyze the electrical properties of the fabricated biosensors. The results were statistically calculated based on 3 independent measurements.

## 3. Results and Discussion

### 3.1. Characterizations of SiNW-FET

**Figure 2:**
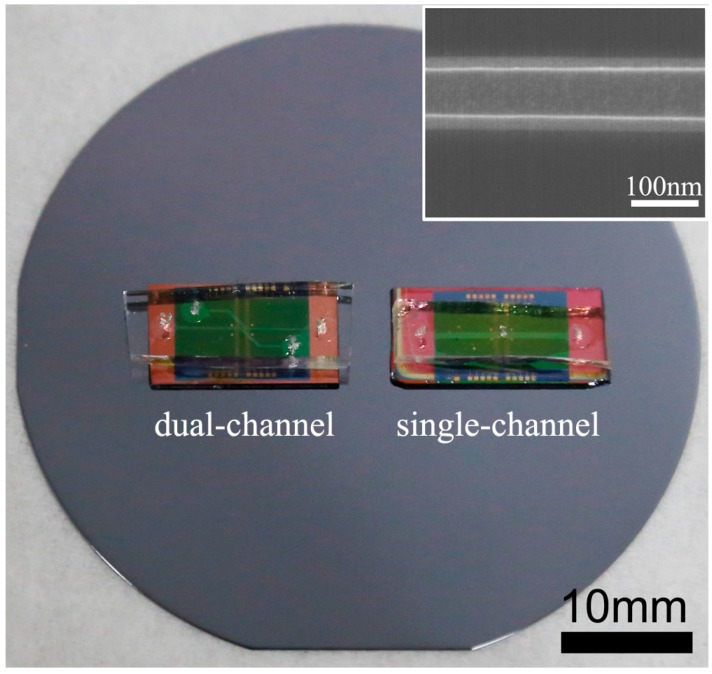
Optical images of fabricated SiNW-FET biosensors with dual-channel and single-channel setups. The inset shows the SEM image of a typical Si nanowire.

The optical image of typical SiNW-FETs integrated with PDMS microfluidic channels is exhibited in Figure 2. The dual-channel SiNW-FET chip containing two individual micro-channels was used to anchor the two antibodies against AFP and CEA to functionalize the NW surface, while the single-channel SiNW-FET chip was used to simultaneously detect the concentrations of AFP and CEA based on the immobilized antibodies. It was mainly composed of an inlet, an outlet and a channel, as well as the modified SiNW with AFP and CEA antibodies. The width of dual-channel PDMS is fabricated to be 200 μm, while it is 500 μm for the single-channel setup. Typical scanning electron microscopy (SEM) image for SiNW is exhibited in the inset of Figure 1, indicating smooth surfaces with a 100-nm width. We can also observe that SiNW exhibited a trapezoid shape caused by the anisotropic etching of silicon by TMAH.

### 3.2. Electrical Transport of the Fabricated SiNW-FET

**Figure 3:**
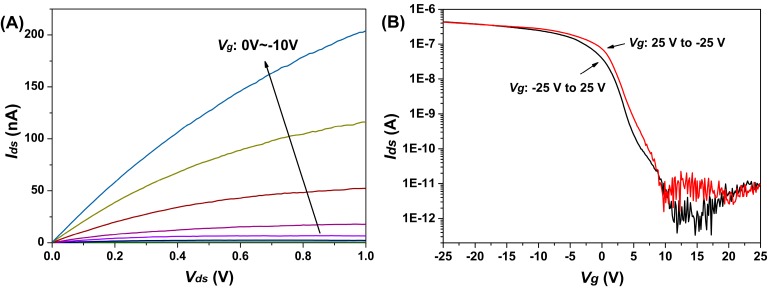
Typical p-type electrical characterization of fabricated SiNW-FETs. (**A**) The *I_ds_* −*V_ds_* output curve depended on the *V_g_* varying from 0 to −10 V; (**B**) The *I_ds_*−*V_g_* transfer curve extracted at *V_ds_* = 1 V.

Semiconductor doping is defined as n-type and p-type, depending on whether the impurity atoms donate electrons to the intrinsic semiconductor (n-type) or accept electrons from the valence band of semiconductor providing holes and increasing the holes’ carrier concentration of the semiconductor (p-type). The output characteristics in Figure 3A indicated the drain current enhanced as the gate voltage changed from 0 V to −10 V. Together with the transfer curve in Figure 3B, the p-type transport behavior of the fabricated SiNW-FET can be observed. The SiNW-FET displayed an excellent performance in terms of high I_on_/I_off_ ratio at about four orders of magnitude. Furthermore, from Figure 3B, little difference can be observed between forward (*V_g_* from −25 V to 25 V) and backward (*V_g_* from 25 V to −25 V) sweep curves, indicating minimal defect-induced charge trapping [19].

### 3.3. Response Behavior of AFP and CEA with SiNW-FET Biosensors

Due to the lower than physiological (pH 7.4) isoelectric points of AFP (4.9) [20] and CEA (3.4) [21], they are negatively charged and accumulate in the holes in the p-type SiNWs, resulting in enhanced conductivity of the SiNW-FETs. Typically, the SiNW-FET functions at different working regimes, including linear or subthreshold regimes. Gao *et al.* studied how the working regimes of SiNW-FETs affected their performances and found that the sensitivity of SiNW-FET can be exponentially enhanced in the subthreshold regime, where the gating effect of molecules immobilized on SiNW surfaces was most effective due to the reduced screening of carriers in SiNWs [22]. In our current study, we followed their results and chose *V_g_* = −8 V, *V_ds_* = 0.5 V for the biosensing measurements, driving the SiNW-FET to function at the subthreshold regime, and the leakage current is measured to be below 150 pA in solutions.

**Figure 4:**
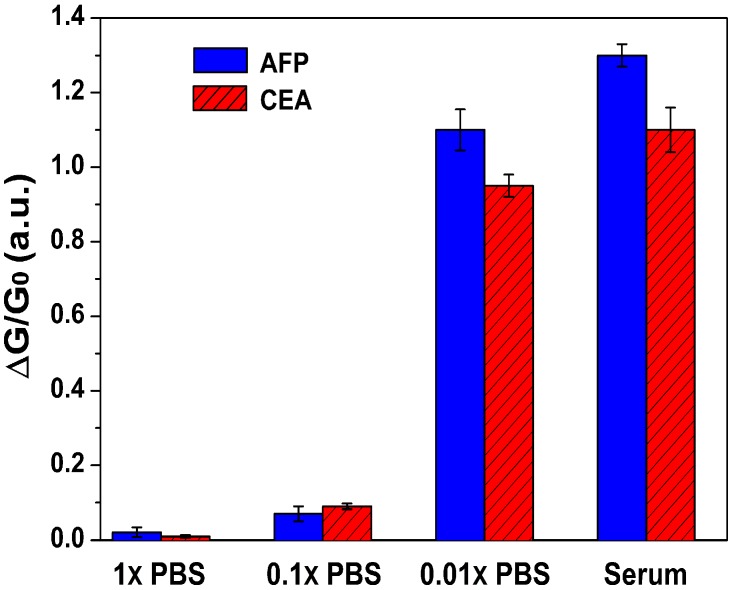
Biosensor responses to 100 pg/mL AFP and CEA in different PBS buffers and desalted serum solution.

We first investigated the response behavior of AFP and CEA with the SiNW-FETs in desalted serum. SiNWs, connected between the source and drain electrodes in the semiconductor channel, are used as the sensing component by immobilizing probes onto the nanowire surfaces to recognize target molecules with a high degree of specificity and affinity. During the past decade, many kinds of SiNW FET biosensors have been explored in the detection of various biological specimens, in which most of them were based on a PBS buffering system. However, a serum system is more clinically relevant, because it contains various biomarkers from different diseases and, at the same time, also ensures the biological activities of these biomarkers. Another important parameter, Debye length, also affects the performance of the FET device. It is defined as the distance over which significant charge separation occurs. In other words, it is the maximum distance over which SiNW carrier concentrations can be impacted by external charges [23]. Debye length is relative to the ionic strength of the solution. A longer Debye length is expected to ensure that less charge is screened with low electrolyte concentrations. Therefore, solutions with low salt concentrations can utilize Debye length sufficiently and reasonably. Since serum commonly contains high levels of salts, which decrease the detection sensitivities of FET biosensors, a desalting process needs to be applied on the pure serum samples to acquire applicable sensitivity [13]. In this work, the sensitivity can be expressed as (*G* − *G_0_*)/*G_0_*. Herein, we further compared the response behaviors of the fabricated biosensors in desalted serum with various concentrations of PBS (1×, 0.1× and 0.01× PBS), which are commonly used in FET-type biosensor designs [24]. Figure 4 illustrates the results of the fabricated biosensors in different solutions. When the sample solutions were injected into the Si nanowire surface, the negatively-charged AFP or CEA antigens bond with their counterparts, *i.e.*, the AFP or CEA antibodies, which were pre-immobilized on the Si surfaces. This kind of antigen- antibody binding would introduce charge changes on the Si nanowires, causing the gating effect to modulate the electrical transport of the semiconducting Si channel. Due to the negative charge, the conductance of the p-type Si channel would be enhanced as a result. We found that the biosensing signals are significantly lower in 1× and 0.1× PBS due to the shielding of the molecular charge by excess ions. When the ionic strength is decreased in 0.01× PBS, significant signal changes can be further observed, consistent with previous reports [13]. Remarkably, when desalted serum was applied, a significantly higher conductance change of the biosensor was acquired. This result indicated that the desalting procedure enhanced the sensitivity of the biosensor, hinting at the preservation of the bioactivity of the AFP and CEA after the desalting procedure, which facilitates the application of the FET-type biosensor in serum systems with clinical relevance.

**Figure 5:**
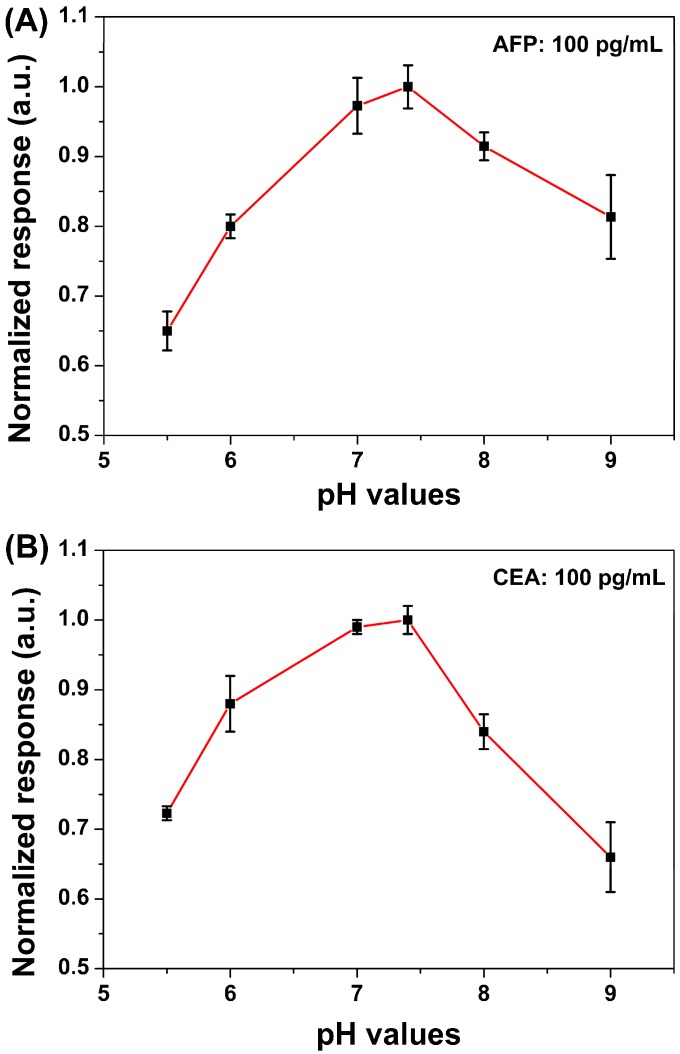
Response behavior to 100 pg/mL AFP (**A**) and CEA (**B**) *versus* different pH values of measured serum.

The sensitivity and conductance of the device are affected by the pH of the solution. Solution pH is optimized by measurements of the relations between pH values and electrical signals. From Figure 5, it can be seen that the response of biosensors first increased with the elevated pH, which can be due to the increased surface charges on AFP and CEA proteins. However, when the solution pH reached relatively high values, the responses decreased accordingly, which might be originated from the limited antigen–antibody interactions in such alkaline environments. In this study, pH 7.4 of serum was used, as it gives rise to the maximal values of signal as shown in Figure 5.

**Figure 6:**
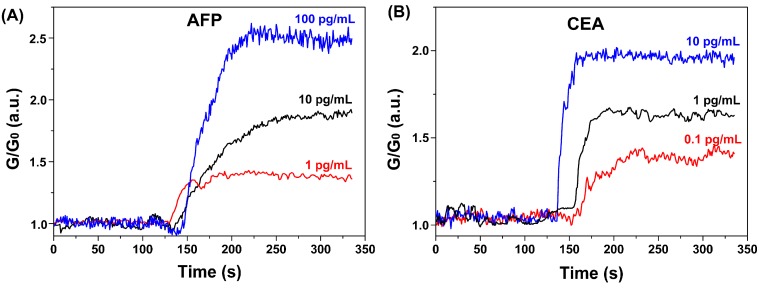
Real-time biosensing behaviors of the fabricated biosensor. Normalized conductance response of SiNW-FET biosensor for AFP (**A**) and CEA (**B**).

Based on the above considerations, we explored the biosensing properties of the fabricated SiNW-FET biosensors towards AFP and CEA proteins. Figure 6 shows the typical biosensing results of simultaneous detection of AFP and CEA at different concentrations. The electrical signals kept constant when blank solutions were applied. After the addition of serum with various concentrations of AFP and CEA, the electrical signals were altered rapidly and became stable within 2 min. Signal strength increased proportionally to AFP and CEA concentrations. The sensing signals also confirmed that negatively-charged proteins enhanced the conductivity of p-type SiNWs. In addition, the sensitivity of the device is often deteriorated by the nonspecific binding of other proteins in the serum, Therefore, BSA was utilized as a blocking reagent on the surface of biosensor to improve the sensitivity, as reported in a previous study [25]. In our control experiments, we observed negligible changes of signals at 100 pg/mL AFP and CEA without BSA blocking, indicating that the nonspecific binding significantly affected the binding between the antigens (AFP and CEA) and their anchored antibodies. Since the total protein concentration of serum can be as high as 40 to 90 mg/mL [18], the above results also indicate the successful BSA blocking of interferences from serum.

**Figure 7:**
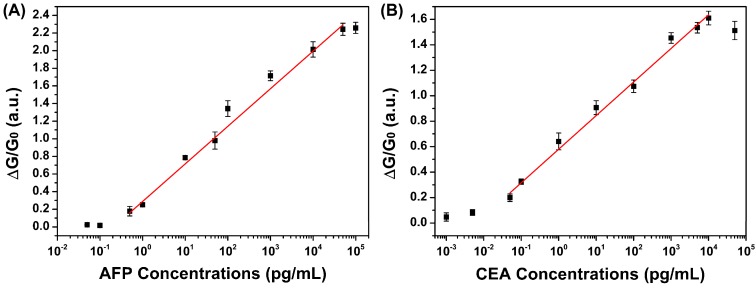
Calibration curves for the response of the fabricated biosensor to AFP (**A**) and CEA (**B**).

AFP and CEA are also expressed in healthy individuals, whereas patients suffering from PHC possess higher levels of AFP and CEA, approximately 10 ng/mL and 2.5 ng/mL, respectively [25,26,27,28]. To assess if our biosensor is able to detect the fractional difference of the concentrations of tumor markers in clinical diagnosis, we calibrated the curves via measuring signals obtained by applying a range of AFP and CEA concentrations. As shown in Figure 7, the linear dynamic ranges for the detection of AFP and CEA in serum are fitted to be 500 fg/mL to 50 ng/mL and 50 fg/mL to 10 ng/mL, respectively. The detection limits for AFP and CEA were measured to be 500 fg/mL and 50 fg/mL with a signal-to-noise ratio of 3, which were significantly lower than those of HRP functionalized Pt hollow nanospheres (90 pg/mL for AFP and 50 pg/mL for CEA) [29], GOD-functionalized carbon nanotubes (2.2 pg/mL for AFP and 1.4 pg/mL for CEA) [30], metal ion-tagged immunocolloidal gold (3.1 pg/mL for AFP and 4.6 pg/mL for CEA) [31] and functionalized graphene (1.33 pg/mL for AFP and 1.64 pg/mL for CEA) [32]. Additional, all of the data points were collected based on three different biosensors, giving a relative standard deviation (RSD) of below 9%, and all of the above results suggest that this device is sufficient to determine the concentration of AFP and CEA at an early stage of PHC with clinical sensitivities.

## 4. Conclusions

In summary, the present study demonstrated that fabricated SiNW-FET biosensors exhibited high sensitivity and a wide range. With the application of PDMS microfluidic channels, we utilized a dual-channel setup to functionalize the surface of the biosensor with both AFP and CEA antibodies on different SiNWs, making it clinically reliable to detect the concentrations of the two tumor makers, AFP and CEA, simultaneously. Our study successfully established a method for the realization of the SiNW FET biosensor with simultaneous detection of multiple biomarkers in the clinical diagnosis of PHC at an early stage.
